# Effects of lesions of the nucleus accumbens core on choice between small certain rewards and large uncertain rewards in rats

**DOI:** 10.1186/1471-2202-6-37

**Published:** 2005-05-28

**Authors:** Rudolf N Cardinal, Nathan J Howes

**Affiliations:** 1 Department of Experimental Psychology, University of Cambridge, Downing Street, Cambridge CB2 3EB, UK

## Abstract

**Background:**

Animals must frequently make choices between alternative courses of action, seeking to maximize the benefit obtained. They must therefore evaluate the magnitude and the likelihood of the available outcomes. Little is known of the neural basis of this process, or what might predispose individuals to be overly conservative or to take risks excessively (avoiding or preferring uncertainty, respectively). The nucleus accumbens core (AcbC) is known to contribute to rats' ability to choose large, delayed rewards over small, immediate rewards; AcbC lesions cause impulsive choice and an impairment in learning with delayed reinforcement. However, it is not known how the AcbC contributes to choice involving probabilistic reinforcement, such as between a large, uncertain reward and a small, certain reward. We examined the effects of excitotoxic lesions of the AcbC on probabilistic choice in rats.

**Results:**

Rats chose between a single food pellet delivered with certainty (*p *= 1) and four food pellets delivered with varying degrees of uncertainty (*p *= 1, 0.5, 0.25, 0.125, and 0.0625) in a discrete-trial task, with the large-reinforcer probability decreasing or increasing across the session. Subjects were trained on this task and then received excitotoxic or sham lesions of the AcbC before being retested. After a transient period during which AcbC-lesioned rats exhibited relative indifference between the two alternatives compared to controls, AcbC-lesioned rats came to exhibit risk-averse choice, choosing the large reinforcer less often than controls when it was uncertain, to the extent that they obtained less food as a result. Rats behaved as if indifferent between a single certain pellet and four pellets at *p *= 0.32 (sham-operated) or at *p *= 0.70 (AcbC-lesioned) by the end of testing. When the probabilities did not vary across the session, AcbC-lesioned rats and controls strongly preferred the large reinforcer when it was certain, and strongly preferred the small reinforcer when the large reinforcer was very unlikely (*p *= 0.0625), with no differences between AcbC-lesioned and sham-operated groups.

**Conclusion:**

These results support the view that the AcbC contributes to action selection by promoting the choice of uncertain, as well as delayed, reinforcement.

## Background

Animals often need to choose between different courses of action on the basis of the eventual rewarding or reinforcing outcomes of those actions. However, the relationship between an action and an outcome is frequently uncertain: animals do not always obtain that for which they work. Therefore, animals must incorporate information on the probability of obtaining different rewards when making decisions about what to do. Little is known of the neural basis of this process. Furthermore, when making decisions under conditions of uncertainty, individuals vary as to how much uncertainty or risk they are willing to tolerate. Formally, individuals differ in how much they 'discount' the value of reinforcers as the uncertainty of the reinforcer increases (i.e. as the probability of the reinforcer declines, or the odds against obtaining the reinforcer increase) [1]. Risk taking is one aspect of the personality trait of impulsivity [2-4] and is a feature of a number of psychiatric disorders, including pathological gambling and certain personality disorders [5-8]. The term 'risk' implies exposure to the possibility of an aversive consequence [9], which may include the possibility of not obtaining an anticipated reward. In the appetitive domain, risk taking is exemplified by the tendency to choose large rewards that are very uncertain, in preference to smaller, certain rewards. Abnormal risk taking may reflect dysfunction of reinforcement learning systems that mediate the effects of uncertain reward or punishment.

The nucleus accumbens (Acb) is one candidate structure that may influence choice involving uncertainty. The Acb responds to anticipated rewards in humans, other primates, and rats [10-17], and is innervated by dopamine (DA) neurons that respond to errors in reward prediction in a manner appropriate for a teaching signal [18-21]. There is clear evidence that the Acb is involved in the processing of delayed reinforcement and its influence upon choice. Damage to the nucleus accumbens core (AcbC) produces impulsive choice in rats [22,23], reducing their ability to choose large, delayed rewards in preference to small, immediate rewards, yet these and other similar lesions do not appear to impair rats' ability to discriminate reward size [23-31]. Furthermore, AcbC lesions impair rats' ability to learn instrumental actions when the outcomes of those actions are delayed [24]. The Acb may also be involved in the processing of uncertain or probabilistic reinforcement. DA neurons that innervate the Acb may fire in a manner related to reward probability [32-34] and the midbrain, the site of the cell bodies of these neurons, responds to stimulus uncertainty in humans [35]. A greater blood flow response is observed in the human Acb during the selection of high-reward/high-risk options, compared to low-reward/low-risk outcomes, in a task where the risk is of not winning [36], with similar activation to high-reward/high-risk option selection in a task where the risk is of losing [37]; this latter activation was correlated with personality measures of harm avoidance. However, these studies are correlative, and it is not known whether the AcbC is causally involved in regulating choice involving uncertain reinforcement.

**Figure 1:**
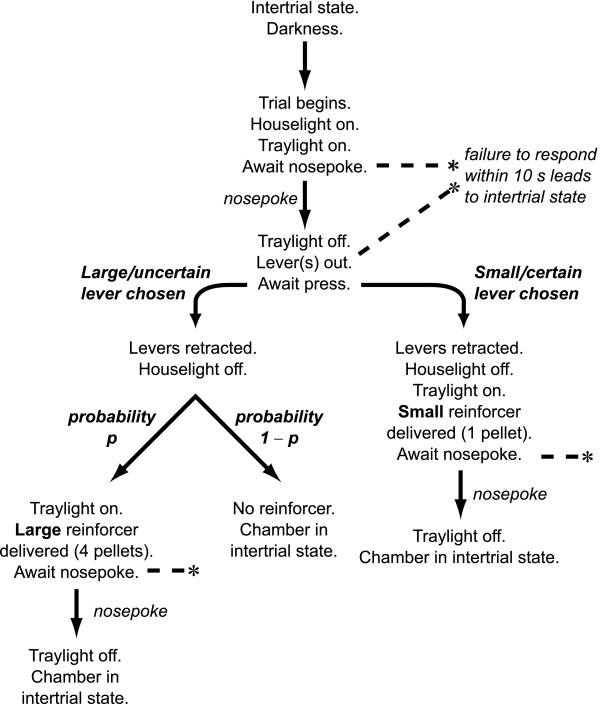
**Task schematic: choice between small, certain and large, uncertain reward**. Probabilistic choice task, based on similar tasks involving choice between delayed reinforcers [73, 74]. Hungry rats regularly chose between two levers. Responding on one lever led to the certain delivery of a small food reward (1 pellet); responding on the other led to a much larger food reward (4 pellets), but this reward was uncertain, and was delivered with a probability (*p*) ranging from 1 to 0.0625. The figure shows the format of a single trial. Trials began at regular intervals (every 40 s). Sessions consisted of 5 blocks. In each block, 16 single-lever trials were given (8 trials for each lever, randomized in pairs), to ensure the animals sampled the options available at that time; these were followed by 10 choice trials. The probability of delivery of the large reinforcer was varied systematically across the session: probabilities for each block were initially 1, 0.5, 0.25, 0.125, and 0.0625, respectively (see Table 1).

In the present study, we sought to examine the contribution of the AcbC to choice involving probabilistic reinforcement in rats. We trained rats on a task in which they could choose regularly between a certain, small reward and an uncertain, large reward in discrete trials (Figure 1) and made excitotoxic AcbC lesions before retesting the rats postoperatively. Preoperatively, the proportion of choice trials in which the large reinforcer was chosen was approximately a linear function of the large-reinforcer probability. Postoperatively, after a transient period in which AcbC-lesioned rats were relatively indifferent between the two reinforcers, compared to sham-operated controls, a stable state emerged in which AcbC-lesioned rats chose the large, uncertain reinforcer less often than shams did. This pattern persisted regardless of whether the large-reinforcer probability increased or decreased across the session. AcbC-lesioned rats and controls continued to exhibit a strong preference for the large reinforcer when it was consistently certain, and a strong preference for the small, certain reinforcer when the large reinforcer was very unlikely; the lesioned and sham-operated groups did not differ from each other in either of these conditions. These results suggest that the AcbC is necessary for the normal impact of unlikely (as well as delayed) reinforcers upon choice.

## Results

### Histology

**Figure 2:**
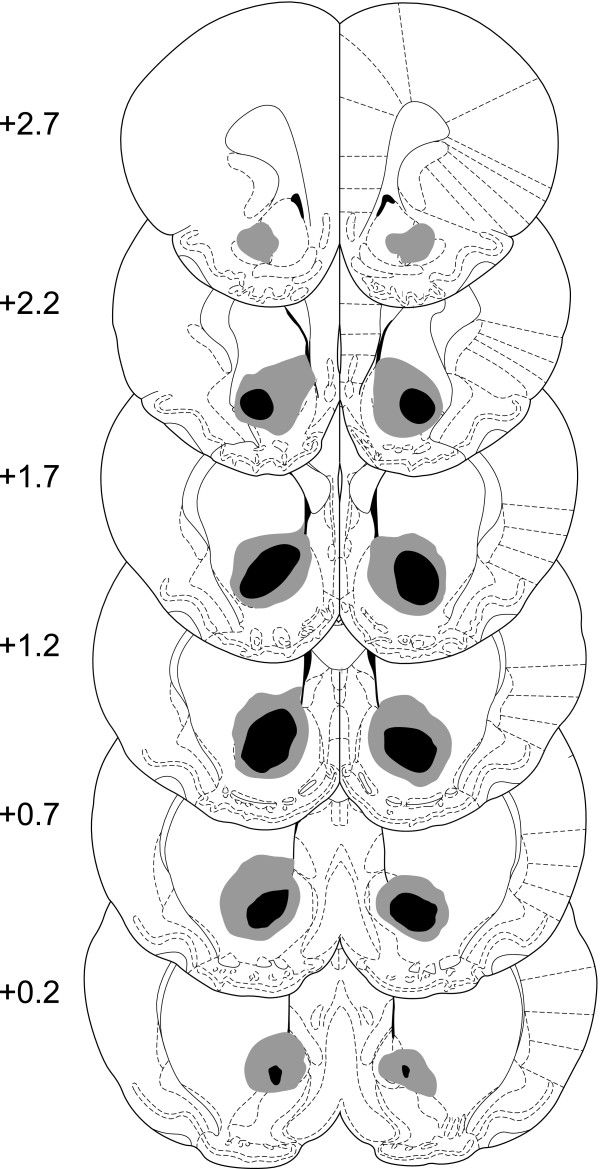
**Schematic of lesions of the nucleus accumbens core**. Black shading indicates the extent of neuronal loss common to all subjects; grey indicates the area lesioned in at least one subject. Coronal sections are (from top to bottom) +2.7, +2.2, +1.7, +1.2, +0.7, and +0.2 mm relative (anterior) to bregma. Diagrams are modified from ref. [136].

There were four postoperative deaths. Histological analysis revealed that the lesions were incomplete or encroached significantly on neighbouring structures in two subjects. These subjects were excluded; final group numbers were therefore 6 (AcbC) and 12 (sham). Lesions of the AcbC encompassed most of the core subregion; neuronal loss and associated gliosis extended in an anteroposterior direction from approximately 2.7 mm to 0.2 mm anterior to bregma, and did not extend ventrally or caudally into the ventral pallidum or olfactory tubercle. Damage to the ventromedial caudate-putamen was occasionally seen; damage to the nucleus accumbens shell (AcbSh) was restricted to the lateral edge of the dorsal shell. Schematics of the lesions are shown in Figure 2. Photomicrographs of lesions with identical parameters have been presented before [24,38,39].

### Preoperative choice

**Figure 3:**
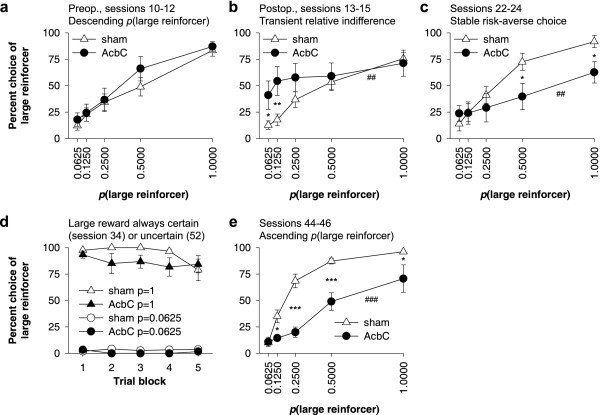
**Choice with probabilistic reinforcement**. **(a) **Preoperative patterns of choice. There were no differences between the groups preoperatively. **(b) **The first three postoperative sessions. Transiently, AcbC-lesioned rats exhibited relative indifference between the two alternatives; their preference did not differ significantly from 50% at any large-reinforcer probability. As a result, AcbC-lesioned rats preferred the large, unlikely reinforcer more than shams did when its probability was 0.0625 and 0.125 (## *p *< .01, lesion × probability interaction; * *p *< .05, ** *p *< .01, comparison to shams at individual probabilities). However, both groups were influenced by the large-reinforcer probability (*p *≤ .004). **(c) **The last three postoperative sessions on the same basic task. By this point, AcbC-lesioned rats preferred the large reinforcer less when its probability was 0.5 or 1 (## *p *< .01, interaction; * *p *< .05, simple effects). Again, both groups were influenced by the large-reinforcer probability (*p *< .001). **(d) **When the 4-pellet reinforcer and the 1-pellet reinforcer were both certain, all groups preferred the 4-pellet reinforcer, and when the 4-pellet reinforcer was always very unlikely (delivered with a probability of 0.0625) and the 1-pellet reinforcer was certain, all groups preferred the 1-pellet reinforcer, with no differences between AcbC-lesioned and sham-operated rats. This indicates that both groups discriminated the reinforcers themselves and discriminated their probability of delivery. **(e) **Choice following further training in which the large-reinforcer probability increased, rather than decreased, across each session. The pattern of choice is similar to c, in that AcbC-lesioned rats were risk-averse compared to shams, i.e. less likely to choose the large, unlikely reinforcer (### *p *< .001, interaction; * *p *< .05 and *** *p *< .001, simple effects). The similarity to c, despite the reversed task order, also indicates that subjects' choice reflected the probabilities in force rather than the order within a session.

The groups remained matched for preoperative choice behaviour following later histological selection (Figure 3a). Choice ratios (percentage choice of the large reinforcer, for each trial block) calculated across sessions 10–12 (see Table 1) were analysed using the model lesion intent_2 _× (large-reinforcer probability_5 _× S). There was a robust effect of probability (*F*_3.3,52.9 _= 70.6, 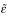 = .826, *p *< .001) but no effect of lesion intent and no lesion intent × probability interaction (*F*s < 1, NS).

### Early postoperative choice

In the initial postoperative period, AcbC-lesioned rats exhibited relative indifference between the two alternatives, choosing the large reinforcer close to 50% of the time at all large-reinforcer probabilities; as a result, AcbC-lesioned rats were more likely than shams to choose the large reinforcer when it was most uncertain (Figure 3b). An analysis of choice ratios calculated across sessions 13–15 was performed using the ANOVA model lesion_2 _× (probability_5 _× S). This revealed a lesion × probability interaction (*F*_3.3,53.5 _= 5.22, 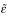 = .836, *p *= .002). Comparison of the two groups at individual large-reinforcer probabilities demonstrated that AcbC-lesioned rats chose the large/uncertain reinforcer more than shams at *p*_reinforcer _= 0.0625 (*p*_statistical _= .02), and at *p*_reinforcer _= 0.125 (*p*_statistical _= .009), but did not differ from shams at reinforcer probabilities of 0.25–1 (*p*_statistical _≥ .158). Nevertheless, simple effects of probability persisted both in shams (*F*_2.8,30.9 _= 32.3, 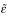 = .702, *p *< .001) and in AcbC-lesioned rats (*F*_4,20 _= 5.37, *p *= .004). Choice at each *p*_reinforcer _was compared to 50% (indifference) using *post hoc *two-tailed one-sample *t *tests, correcting *p*_statistical _values using the Šidák correction for 5 comparisons. For shams, choice differed significantly from 50% at large-reinforcer probabilities of 0.0625 (when choice of the large reinforcer was less than 50%), 0.125 (less than 50%), and 1 (greater than 50%) (corrected *p*_statistical _≤ 0.007), but for AcbC-lesioned rats, choice did not differ significantly from 50% at any large-reinforcer probability (corrected *p*_statistical _≥ 0.81).

### Final postoperative choice

By the final three sessions of the basic task (sessions 22–24; see Table 1), the pattern of choice in AcbC-lesioned rats had changed (Figure 3c). Once more, an analysis of choice ratios using the model lesion_2 _× (probability_5 _× S) revealed a lesion × probability interaction (*F*_2.9,46.4 _= 5.78, 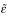 = .726, *p *= .002). By now, however, AcbC-lesioned rats did not differ from shams with reinforcer probabilities of 0.0625-0.25 (*p*_statistical _≥ .386) but chose the large reinforcer *less *than shams when its probability was 0.5 (*p*_statistical _= .037) and 1 (*p*_statistical _= .015). As before, effects of probability persisted both in shams (*F*_2.3,24.9 _= 49.5, 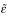 = .565, *p *< .001) and in AcbC-lesioned rats (*F*_4,20 _= 9.45, *p *< .001).

### Choice when both reinforcers were certain, or both uncertain

When the large and small reinforcers were both delivered with certainty, AcbC-lesioned and sham-operated rats strongly preferred the large reinforcer; when the small reinforcer was certain and the large reinforcer was consistently unlikely (*p*_reinforcer _= 0.0625), all rats strongly preferred the small reinforcer (Figure 3d). There were no group differences in either case. This indicates that both AcbC-lesioned and sham-operated rats successfully discriminated the large reinforcer from the small reinforcer, and discriminated the certain large reinforcer from the uncertain large reinforcer. Choice ratios from the final sessions of training in these two conditions (sessions 34 and 52; see Table 1) were analysed using the model lesion_2 _× (trial block_5 _× S). In the 'certain' condition (session 34), there was no effect of lesion (*F*_1,15 _= 2.54, *p *= .132), no lesion × block interaction (*F *= 1.42, NS), and no effect of trial block (*F*_1.5,21.9 _= 2.12, 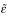 = .365, *p *= .154). Similarly, in the 'uncertain' condition (session 52), there was no effect of lesion (*F *= 1.35, NS), no lesion × block interaction (*F *= 1.31, NS), and no effect of trial block (*F *< 1, NS).

### Choice with ascending probabilities

After rats had been trained with the large-reinforcer probability *increasing *across the session, choice behaviour was similar to that with the decreasing-probability version of the task used initially, with AcbC-lesioned rats choosing the large/uncertain reinforcer less often than shams (Figure 3e; compare Figure 3c). Choice ratios from sessions 44–46 (see Table 1) were analysed using the model lesion_2 _× (probability_5 _× S). As before, there was a lesion × probability interaction (*F*_4,64 _= 9.29, *p *< .001), in addition to main effects of lesion (*F*_1,16 _= 19.5, *p *< .001) and probability (*F*_4,64 _= 95.6, *p *< .001), and there were strong effects of probability for both AcbC-lesioned rats (*F*_1,20 _= 20.7, *p *< .001) and shams (*F*_3.1,34.4 _= 119.6, 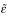 = .781, *p *< .001). AcbC-lesioned rats differed from shams at reinforcer probabilities of 0.125 (*p*_statistical _= .033), 0.25 (*p*_statistical _< .001), 0.5 (*p*_statistical _< .001), and 1 (*p*_statistical _= .013), but not at *p*_reinforcer _= 0.0625 (*p*_statistical _= .881).

### Postoperative choice: analysis by experienced probability

**Figure 4:**
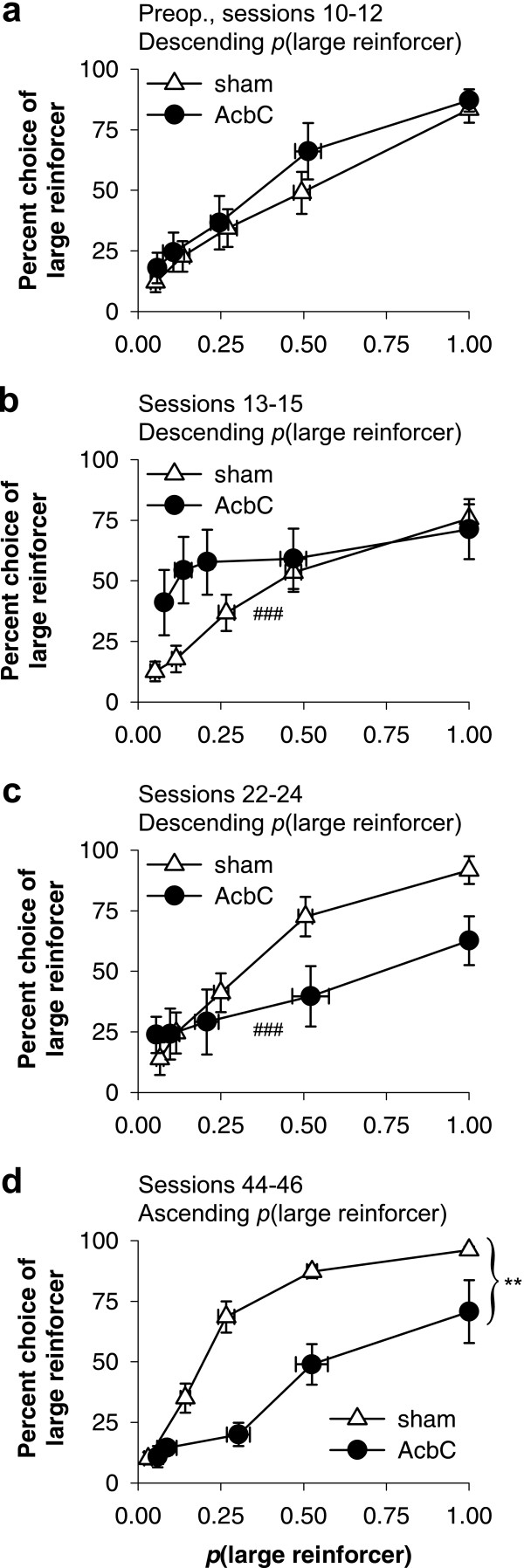
**Choice, by experienced probability**. Choice, replotted by experienced (as opposed to programmed) large-reinforcer probabilities. Panels **a-d **correspond to panels **a-c/e **of the previous figure. The statistical patterns of choice remained the same (### *p *< .001, lesion × experienced delay interaction; ** *p *< .01, main effect of lesion).

Since the task was genuinely probabilistic, and not pseudorandom, it is possible that the probabilities experienced by subjects differed from the programmed probabilities (although experienced probabilities inevitably tend towards programmed probabilities as the number of trials increases). For example, one subject choosing an uncertain reinforcer at *p*_reinforcer _= 0.5 for 10 trials might experience 3 rewarded and 7 unrewarded trials (an experienced probability of 0.3), while another might experience 6 rewarded and 4 unrewarded (experienced *p*_reinforcer _= 0.6). To establish whether such effects accounted to any degree for the pattern of choice observed in AcbC-lesioned and sham-operated rats, choice was re-analysed for four sets of sessions (preoperative sessions 10–12, early postoperative baseline sessions 13–15, late postoperative baseline sessions 22–24, and sessions 44–46 at the end of training on the increasing-probability version of the task; see Figure 4a–d, compared to the corresponding programmed-probability versions in Figure 3a–c,e). In each case, choice ratios were analysed using the model lesion_2 _× (experienced probability_cov _× S), with the factor × covariate term included in the model. Experienced probabilities were calculated for all trial types (forced and choice trials), across the sessions concerned.

These analyses confirmed the pattern of results obtained on the basis of programmed probabilities. For the preoperative sessions, as expected, there was a main effect of experienced probability (*F*_1,54 _= 319.1, *p *< .001) but no significant terms involving lesion intent (*F*s < 1, NS). For the baseline (decreasing-probability) task, both early (sessions 13–15) and late (sessions 22–24) in the postoperative testing, there was a lesion × experienced probability interaction (early: *F*_1,54 _= 25.7, *p *< .001; late: *F*_1,54 _= 20.8, *p *< .001). For the increasing-probability schedule (sessions 44–46), there was no lesion × experienced probability interaction (*F*_1,54 _= 1.80, *p *= .185) but there was a main effect of lesion (*F*_1,16.0 _= 9.36, *p *= .007).

### Indifference probabilities

Choice ratios from sham-operated rats on sessions 22–24 (the final 3 postoperative sessions on the basic task; see Table 1) were analysed using four different linear predictors, based either on the probability of delivery of a large reinforcer (given choice of the Large lever), or of the odds against delivery of a large reinforcer, calculated as *odds against *= (1 - *p*)/*p*. This established that choice patterns were predicted best, in linear fashion, by experienced probabilities (within-subject predictor allowing different slopes for each subject, *r*^2 ^= 0.85) and programmed probabilities (*r*^2 ^= 0.84), rather than by experienced odds (*r*^2 ^= 0.61) or programmed odds (*r*^2 ^= 0.67). Since optimal behaviour would give choice that was a step function of probability (i.e. it is optimal to choose the small/certain lever whenever the 4-pellet reinforcer is delivered with *p*_reinforcer _< 0.25 and to choose the large/uncertain lever whenever *p*_reinforcer _> 0.25), a single-parameter continuous function approximating a step function was also used to predict subjects' choice [the logistic function *y *= 100/*e*^-(*x*-*m*)/*b *^with *y *as the percentage choice of the large reinforcer, *x *as the programmed probability, *b *= 0.01 as an approximation to *b *= 0 and *m *as the free parameter], but this gave a poor fit (*r*^2 ^calculated as SS_model_/SS_total _for a nonlinear fit: mean *r*^2 ^= 0.26; note that individual values of *r*^2 ^can fall outside the range [0,1] when calculated this way for nonlinear models) [40]. Consequently, since choice was best described as a linear function of probability, indifference probabilities were calculated for sham-operated and AcbC-lesioned rats, namely the probability at which rats were equally likely to choose the small/certain and large/uncertain reinforcers. These were calculated via a linear regression of probability on choice (i.e. a regression in which probability was predicted from choice). This method has the potential to produce nonsensical probabilities for individual rats (if, for example, an individual's curve does not go both above and below the 50% choice point in a given set of sessions) but is nonetheless useful for group comparison. Experienced large-reinforcer probabilities (across all types of trials) were used, rather than programmed probabilities, though the pattern of results presented below was not altered by the use of programmed probabilities instead.

**Figure 5:**
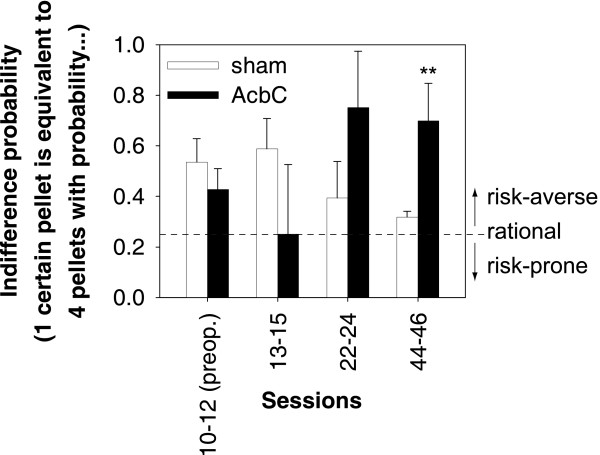
**Indifference probabilities**. Subjects' behaviour was analysed using a linear regression technique (see text for method of calculation) to estimate the large-reinforcer probability at which they were indifferent between a 4-pellet uncertain large reinforcer and a 1-pellet certain small reinforcer. Rational choice, and optimal choice in this task, would be an indifference probability of 0.25 (that is, it is rational to be indifferent between a certain 1-pellet reinforcer and a 4-pellet reinforcer delivered with a probability of 0.25), shown by the dotted line. Lower indifference probabilities imply risk-prone behaviour; higher indifference probabilities imply risk-averse behaviour. Preoperative and successive postoperative indifference probabilities are shown for AcbC-lesioned and sham-operated control rats (** *p *< .01, difference from controls).

The main finding was that by the end of testing, AcbC-lesioned rats had higher indifference probabilities (0.70) than sham-operated rats (0.32) (Figure 5) – that is, while sham-operated rats behaved as if indifferent between a 1-pellet certain reinforcer and a 4-pellet reinforcer delivered with probability 0.32 (mathematically, an expected number of pellets of 0.32 × 4 = 1.28), AcbC-lesioned rats behaved as if indifferent between a 1-pellet certain reinforcer and a 4-pellet reinforcer delivered with probability 0.70 (an expected number of pellets of 2.8). That is, AcbC-lesioned rats appeared to exhibit risk aversion by the end of testing. The full analysis was as follows. Preoperatively (sessions 10–12), indifference probabilities were 0.43 ± 0.08 (AcbC) and 0.54 ± 0.09 (sham); these did not differ (*F *< 1, NS). In the initial postoperative period (sessions 13–15), indifference probabilities were numerically lower in the lesioned group, being 0.25 ± 0.28 (AcbC) and 0.59 ± 0.12 (sham), but indifference probabilities were highly variable in both groups and these did not differ (*F*_1,16 _= 1.76, *p *= .204). In the later postoperative period (sessions 22–24), indifference probabilities were higher in the lesioned group, being 0.75 ± 0.22 (AcbC) and 0.39 ± 0.15 (sham), though again these did not differ significantly (*F*_1,16 _= 1.90, *p *= .187). In the increasing-probability version of the task (sessions 44–46), indifference probabilities were again higher in the lesioned group, being 0.70 ± 0.15 (AcbC) and 0.32 ± 0.02 (sham). By this stage the difference was highly significant (*F*_1,16 _= 12.6, *p*_statistical _= .003), even if corrected for four comparisons (*p*_statistical _= .012) using the Šidák correction.

### Omissions and latencies

Omissions were infrequent and not influenced by reinforcer probability or the lesion. Omission data from the final postoperative baseline sessions (sessions 22–24) were analysed. Overall, omissions (either failures to initiate a trial or to respond to an initiated trial) across all trial types occurred at a rate of 2.9 ± 0.9 % (sham) and 5.5 ± 1.9 % (AcbC). Omissions on choice trials for the same sessions were analysed using the model lesion_2 _× (probability_5 _× S). There were no effects of lesion (*F*_1,16 _= 1.95, NS) or probability (*F*_1.6,25.2 _= 2.56, 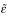 = .394, *p *= .107), and no interaction (*F *= 1.04, NS). Almost all omissions were failures to initiate a trial (shams 0.9% of choice trials, AcbC 4.4%) rather than failures to respond once a trial had been initiated (shams 0.06% of choice trials, AcbC 0%).

Initiation latencies on choice trials for sessions 22–24 were analysed in the same manner. They were not affected by the lesion (*F *< 1, NS), nor by the large-reinforcer probability (*F*_4,64 _= 1.41, NS), and there was no lesion × probability interaction (*F *< 1, NS).

Response latencies were not affected by the lesion, but were affected both by the time in the session, with responding tending to get slower as the session progressed, and by the likelihood of obtaining a large reinforcer, with responding tending to get faster as large-reinforcer delivery became more likely. Response latencies on choice trials for sessions 22–24 were analysed using the model lesion_2 _× (trial block_5 _× choice_2 _× S). Response latencies varied across trial blocks: response latencies were initially 0.82 s (in the first trial block, when the large-reinforcer probability was 1) and slowed to 1.1 s (in the last trial block, when the large-reinforcer probability was 0.0625) (*F*_3.1,25.1 _= 2.97, 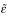 = .785, *p *= .049). Latencies were not affected by the lesion, or the lever being chosen, and there were no interactions (maximum *F *was for response: *F*_1,8 _= 2.96, *p *= .124). To establish whether these effects were due to the large-reinforcer probability, or to progressive satiation or the passage of time, data from sessions 44–46 were also analysed, because in these sessions the large-reinforcer probability *increased *within the session. This time, there was a response × trial block interaction (*F*_4,28 _= 6.44, *p *= .001), with no other terms significant (*F*s < 1, NS). Responding on the small/certain lever initially took 0.71 s in the first trial block and slowed to 0.95 s in the last trial block (*F*_2.3,24.8 _= 3.58, 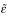 = .564, *p *= .038), but responding on the large/uncertain lever initially took 0.97 s (in the first trial block, when the large-reinforcer probability was 0.0625) and speeded up to 0.79 s (in the last trial block, when the large-reinforcer probability was 1) (*F*_3.5,38.8 _= 3.222, 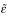 = .883, *p *= .027).

The lesion did not affect the latency to collect reward. Food collection latencies on rewarded trials were analysed across sessions 22–24, this time including both forced and choice trials to enable an analysis by response and probability. The model lesion_2 _× (probability_5 _× response_2 _× S) was used; this revealed main effects of response (*F*_1,13 _= 13.8, *p *= .003) and probability (*F*_2.5,32.8 _= 3.53, 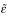 = .631, *p *= .031), but no other significant terms (maximum *F *was for lesion × response, *F*_1,13 _= 3.94, *p *= .069). Collection was faster following delivery of the large reinforcer than the small (4.1 versus 5.3 s, respectively), and got slightly slower across the session (4.4 s in the first trial block and 4.9 s in the last).

### Amount of food obtained

**Figure 6:**
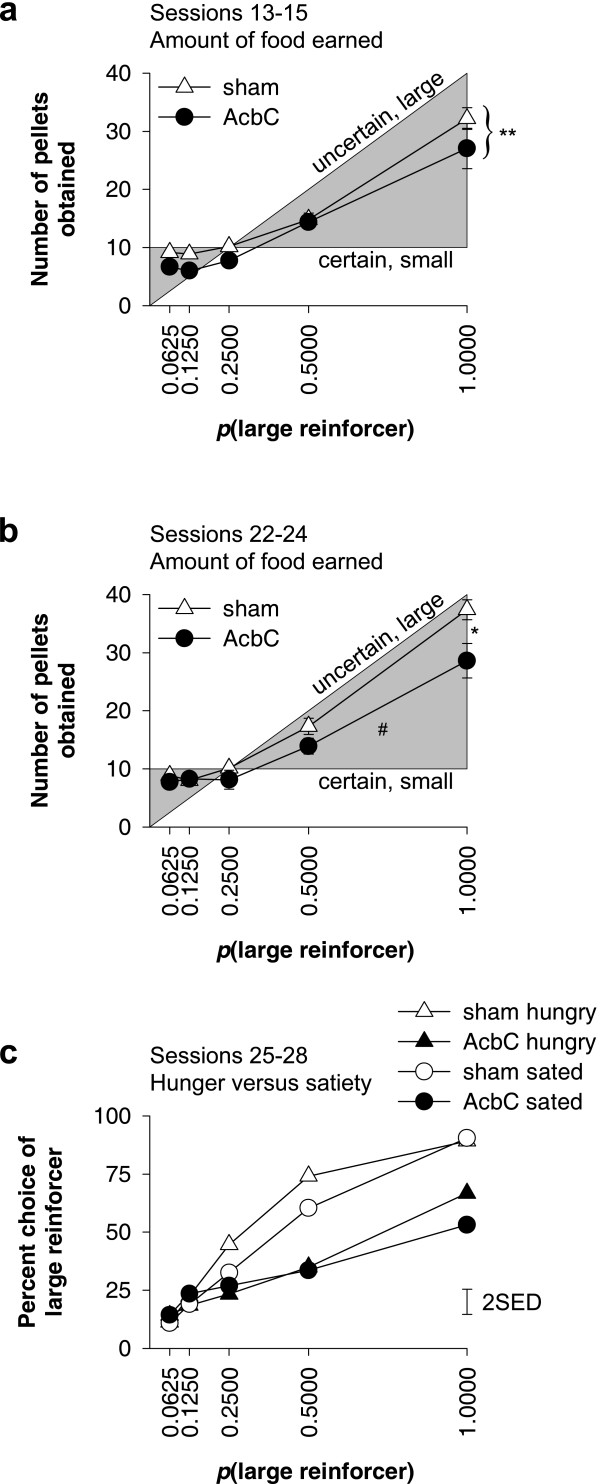
**Amount of food obtained, and effects of satiety on choice**. **(a) **Number of pellets obtained in each trial block; average of the first three postoperative sessions, 13–15 (** *p *< .01, main effect of lesion). The grey area indicates the expected range of options available to a rat making no omissions: consistent responding on the lever delivering the small, certain reward of a single pellet yields 10 pellets per trial block (horizontal border of the grey area); consistent responding on the lever delivering the large, uncertain reward yields an expected number of pellets that varies with the probability in force (as shown by the diagonal border of the grey area). Optimal behaviour, to maximize the expected amount of food, is to choose the small/certain lever when the large (4-pellet) reinforcer probability is less than 0.25 and to choose the large/uncertain lever when this probability exceeds 0.25. **(b) **As for (a), but showing data from the final baseline postoperative sessions, 22–24 (# *p *< .05, lesion × probability interaction; * *p *< .05, simple effect of lesion). **(c)** Effects on choice of alternating subjects between states of hunger and satiety. The error bar is twice the SED for the three-way (lesion × hunger × probability) interaction.

AcbC-lesioned rats obtained less food as a result of their choices (Figure 6a,b). An analysis of the average number of pellets obtained on choice trials in sessions 13–15 using the model lesion_2 _× (probability_5 _× S) revealed a main effect of lesion (*F*_1,16 _= 8.69, *p *= .009), as well as an effect of probability (*F*_1.9,30.1 _= 97.5, 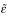 = .470, *p *< .001), but no interaction (*F *< 1, NS). A similar analysis of the final baseline postoperative sessions 22–24 revealed a lesion × probability interaction (*F*_2.1,33.8 _= 3.29, 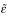 = .529, *p *= .047) in addition to main effects of lesion (*F*_1,16 _= 14.2, *p *= .002) and probability (*F*_2.1,33.8 _= 122.2, 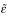 = .529, *p *< .001). However, the only probability at which groups significantly differed was *p *= 1 (statistical *p *= .014); when the large reinforcer probability was 0.0625-0.5, the two groups did not differ in the amount of food obtained (*p*_statistical _≥ .129).

### Effects of hunger and satiety on choice

Alternating between hunger and satiety had no substantial effects on choice (Figure 6b). Choice ratios for sessions 25–28 were analysed using the model lesion_2 _× (hunger_2 _× probability_5 _× S). As before, a main effect of probability (*F*_2.0,32.6 _= 38.4, 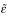 = .510, *p *< .001) and a lesion × probability interaction (*F*_2.0,32.6 _= 4.29, 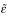 = .510, *p *= .022) were present; in addition, there was a marginally significant lesion × hunger × probability interaction (*F*_4,64 _= 2.51, *p *= .05). However, an effect of hunger was not detectable in either group alone, either for shams (hunger: *F*_1,11 _= 2.45, NS; hunger × probability: *F*_4,44 _= 2.18, NS) or for AcbC-lesioned rats (hunger: *F *< 1, NS; hunger × probability: *F*_4,20 _= 1.79, NS). Similarly, the differences between groups persisted both in the hungry (lesion × probability: *F*_2.6,41.7 _= 3.66, 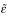 = .652, *p *= .024) and the sated (lesion × probability: *F*_2.5,39.3 _= 4.24, 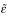 = .615, *p *= .016) conditions.

### Locomotor activity and body mass

**Figure 7:**
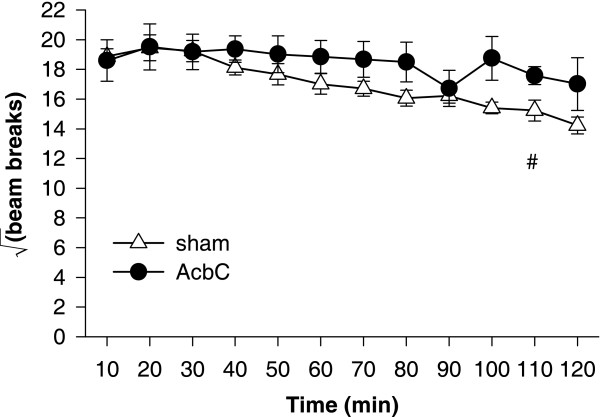
**Locomotor activity in a novel environment**. AcbC-lesioned rats were hyperactive compared to sham-operated controls, being slower to habituate to a novel environment. Analysis using the model lesion_2 _× (bin_12 _× S) revealed a lesion × bin interaction (*F*_8.3,133.4 _= 2.20, 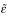 = .758, # *p *= .029), reflecting a difference in habituation between the groups, and a main effect of bin (*F*_8.34,133.4 _= 9.02, *p *< .001), reflecting habituation, though there was no main effect of lesion (*F*_1,16 _= 2.24, *p *= .154).

AcbC-lesioned rats were hyperactive and slower to habituate to a novel environment (Figure 7). AcbC-lesioned rats also gained less mass postoperatively. At the time of surgery, the groups did not differ in mass (shams, 357 ± 4 g; AcbC, 362 ± 6 g; *F *< 1, NS), but at the end of the experiment AcbC-lesioned rats weighed less than shams (shams, 421 ± 7 g; AcbC, 358 ± 10 g; lesion × time, *F*_1,16 _= 80.1, *p *< .001; simple effect of lesion at final time point: *F*_1,16 _= 24.5, *p *< .001). Both effects are consistent with previous results: AcbC-lesioned rats are known to exhibit locomotor hyperactivity [22,24,38,41] and to weigh less than sham-operated controls [22,24,41,42]. They also eat the food used as the maintenance diet in the present study more slowly than sham-operated controls, and eat less of it in a given time, but do not differ in consumption of the sucrose pellets used as reinforcers in the present study [22,39]. It is not known whether there are metabolic differences in AcbC-lesioned rats above and beyond the tendency to eat somewhat less and to be hyperactive (though see [43]). However, differences in mass between AcbC-lesioned and sham-operated rats are also apparent when they have been fed *ad libitum *ever since the lesion was made, with AcbC-lesioned rats weighing ~88% as much as sham-operated controls in this situation [39], much as in the present study (85%). This suggests that the food deprivation regimen maintained the proportional relationship between actual and free-feeding mass similarly in sham-operated and AcbC-lesioned rats.

## Discussion

These results suggest that the AcbC contributes to the selection of uncertain rewards. AcbC-lesioned rats exhibited risk-averse choice: they chose large, uncertain rewards less than sham-operated controls when offered a smaller, certain alternative, even though they showed a strong and unaltered preference for large rewards over small rewards, and for certain rewards over uncertain rewards. By the end of testing, the control group behaved as if indifferent between a single certain food pellet and four pellets delivered with *p *= 0.32 (close to the probability of 0.25 that would represent rational indifference), while the AcbC-lesioned group behaved as if indifferent between a single certain pellet and four pellets delivered with *p *= 0.70.

Though these results establish that the lesions used in this study caused this pattern of behaviour, the precise mechanism by which this occurs is unknown: for example, it is possible that the damage caused to structures adjacent to the AcbC, though limited, played a role in this pattern of choice, or that adaptations in other structures consequent upon the lesion were important in the behavioural effects (particularly given that risk aversion was not apparent immediately but emerged with further time and postoperative experience with the task).

### Choice in normal subjects

The dominant model of uncertainty or probability discounting [1,44-46] suggests that subjects calculate a value for each reinforcer, according to its size and other parameters, and discount this by multiplying it by 1/(1+*Hθ*), where *θ *represents the odds against obtaining the reinforcer, *θ *= (1 - *p*)/*p*, and *H *represents an odds discounting parameter that is specific to the individual subject but stable over time for that subject. In this model, value is a hyperbolic function of the odds *θ*; such a hyperbolic function is supported by empirical research, at least in humans [44,45,47-50]. The present task is not well suited to evaluating such a quantitative model, since in discrete-trial schedules it is often the case that animals maximize, or allocate most of their choices to whichever option is the more favourable [51]. However, the behaviour of normal subjects here can be evaluated as to its optimality. In the present task, neither risk aversion nor risk taking is optimal if carried to extremes. Optimal behaviour, to maximize the expected amount of food, is to choose the small/certain lever when the large (4-pellet) reinforcer probability is less than 0.25, to choose the large/uncertain lever when the probability exceeds 0.25, and to be indifferent at *p *= 0.25 (i.e. to exhibit a step function in choice). Shams' choice of the large reinforcer behaviour was better described by a linear function of the large-reinforcer probability than by such a step function. Nevertheless, shams' behaviour was reasonably close to the optimal in the most obvious way to measure optimality, namely the amount of food obtained (Figure 6b).

### Effects of AcbC lesions in terms of conditioning processes

AcbC-lesioned rats chose the large, uncertain reinforcer less often than shams did, but only when a smaller certain reinforcer was available as an alternative; that is, they exhibited risk-averse choice. A number of simple explanations of the present results may be ruled out. For example, it is unlikely that the pattern of choice exhibited by AcbC-lesioned rats can be explained in terms of perseveration, within a session, on the initially-optimal lever. It might be that animals that perseverated on the lever delivering the small, certain reinforcer, because that lever was initially optimal, would appear to exhibit risk-averse choice in sessions in which the large-reinforcer probability increased across the session (Figure 3e), but this could not explain the same pattern of choice in sessions in which the same lever was initially suboptimal, i.e. when the large-reinforcer probability decreased across the session (Figure 3c). Furthermore, although AcbC lesions are known to affect processes through which Pavlovian conditioned stimuli (CSs) affect behaviour, including Pavlovian-instrumental transfer (PIT), autoshaping, and conditioned reinforcement [38,52-57], there was no Pavlovian CS that was differentially associated with uncertain as opposed to certain reinforcement in this task, so these effects cannot explain the present results. It might be that the AcbC lesion impaired subjects' knowledge of the instrumental action-outcome contingency specifically for the uncertain outcome. There is some debate about the role of the AcbC in instrumental conditioning (see [43,58,59]) and goal-directed action, a subset of instrumental conditioning [58,60,61]. Manipulation of the AcbC can certainly affect instrumental learning [62-65]. However, the AcbC is not required for simple instrumental conditioning: rats with AcbC lesions acquire lever-press responses on fixed-ratio-1 schedules at supernormal levels [24], and rats with Acb or AcbC lesions are fully sensitive to changes in the action-outcome contingency [25,53,66]. However, when acquiring a sequence of random ratio schedules, AcbC-lesioned rats respond somewhat less than sham-operated controls [66], while lesions of the whole Acb made rats respond slightly, though not significantly, less on a similar sequence of random ratio schedules [53]. Random ratio schedules clearly involve probabilistic reinforcement, so these results are consistent with the possibility that the present impairment shown by AcbC-lesioned rats in choosing large, unlikely rewards is due to impaired instrumental conditioning when the outcome is uncertain – and, conversely, that the impairment in simple instrumental learning seen previously [66] was specifically a result of the reward uncertainty inherent in a random ratio schedule, given that AcbC-lesioned rats learn instrumental responses normally or supernormally with certain immediate reinforcement [24]. It is also possible that AcbC-lesioned rats represent the instrumental contingency normally with uncertain reward, but simply value the uncertain outcome less and respond less for it accordingly, as discussed next.

### Effects of AcbC lesions in terms of probability discounting and reinforcer magnitude sensitivity

Since the present study required rats to choose between small, certain and large, uncertain rewards, an effect of the lesion to alter the perception of relative reward magnitude might affect choice, just as an alteration in the perception of reward probability might. For example, altering the absolute magnitudes of the reinforcers can affect choice involving probabilistic reinforcement [67,68], as would be predicted if reinforcer 'value' is not simply a linear function of physical magnitude [1]. Specifically, the present results (a tendency for AcbC-lesioned rats to choose the small, certain reinforcer more than shams) could be explained by 'risk aversion' (increased or steeper uncertainty/odds/probability discounting), or if the difference between 1 and 4 pellets was perceived to be smaller by AcbC-lesioned subjects than by shams (due to reduced discrimination between the two reinforcer magnitudes, or perhaps with a normal ability to tell the two apart but with an altered perception of relative value). For example, if a normal subject assigned values of 1 and 4 to the reinforcers, and a lesioned subject assigned values of 1 and 3 to the same reinforcers, then the lesioned subject would be less likely than the sham to choose the large reinforcer when it was made uncertain, even without any primary abnormality in the processing of probability. At first glance, this interpretation would appear to be supported by the observation that AcbC-lesioned rats chose the large reinforcer somewhat less often than shams when it was certain, as well as when it was uncertain. However, several lines of evidence suggest this explanation is not the correct one. When the large and the small reinforcers were both made consistently certain, there were no differences between AcbC-lesioned rats and controls (Figure 3d). Furthermore, other evidence indicates that AcbC lesions do not impair reinforcer magnitude discrimination or the perception of relative reinforcer value. Excitotoxic lesions of the whole Acb do not prevent rats from detecting changes in reward value (induced either by altering the concentration of a sucrose reward or by changing the deprivational state of the subject) [25]. Such lesions also do not impair rats' ability to respond faster when environmental cues predict the availability of larger rewards [26], and nor does inactivation of the Acb with local anaesthetic or blockade of AMPA glutamate receptors in the Acb [27,69]; the effects of intra-Acb NMDA receptor antagonists have varied [69-71]. AcbC-lesioned rats can still discriminate large from small rewards [23,28]. Similarly, DA depletion of the Acb does not affect the ability to discriminate large from small reinforcers [29-31], and systemic DA antagonists do not affect the perceived quantity of food as assessed in a psychophysical procedure [72]. Furthermore, a recent study found evidence that AcbC-lesioned rats may even show somewhat enhanced reinforcer magnitude discrimination (or an exaggerated perception of relative value) [24]. Given that reinforcer magnitude discrimination appears to be unimpaired, at worst, by AcbC lesions, the observation in the present study that AcbC-lesioned rats chose the large reinforcer somewhat less often than controls in the task in which large-reinforcer probabilities changed throughout the session is more likely to be explained by within-session generalization [23,73,74] – i.e. that avoidance of the large reinforcer during trial blocks when it was uncertain generalized to trial blocks when it was certain. Together, these findings suggest that the present results are best explained as an effect of AcbC lesions to increase the rate of uncertainty/odds/probability discounting – effectively, a tendency to behave as if an uncertain outcome were less likely than it really is.

### Probability versus delay discounting

It is known that AcbC lesions affect choice and learning involving delayed reinforcement [22-24]. It has been suggested that delay (or temporal) discounting, the process by which delayed reinforcers lose value, and probability (or odds) discounting, the process by which uncertain reinforcers lose value, reflect the same underlying process [44,45,75-81]. For example, in the present task, choosing the uncertain large reinforcer five times but only obtaining it on the fifth response might be seen as equivalent to a very long delay, on average, between choice of the large reinforcer and its eventual delivery. Alternatively, delays may be seen as entailing the ecological risk of losing the reward during the delay. The failure of AcbC-lesioned rats to choose an uncertain reinforcer (risk aversion, as seen in the stable phase of the present results) and their failure to choose a delayed reinforcer may therefore be explained in the same way. However, there is evidence that time and probability discounting are different and dissociable processes [1,46,82]. Most simply, it is not surprising that currency inflation affects human decisions involving delayed but not probabilistic financial reward [83]. Moreover, the absolute magnitude of rewards can have different effects on delayed and probabilistic discounting [46,84,85]. A study looking at human choices in a gambling task found that individuals' propensity to choose rapidly (one, perhaps motoric, measure of delay aversion) and their propensity to bet large amounts of money on uncertain outcomes (a measure of risk taking) represented independent factors [86]. Some studies have found abnormal delay discounting, but not uncertainty discounting, in drug addicts [82,87-89], while gamblers have been observed to discount probabilistic rewards less steeply than controls (i.e. to take risks) without showing differences in delay discounting [8].

### Implications for AcbC function and impulsivity

Impulsivity is multifaceted, reflecting – at the least – individual differences in distinct and dissociable processes involving information gathering, the selection of outcomes, and the inhibition of motor actions [90]. Furthermore, as discussed above, delay discounting and probability discounting may also reflect separate processes. Damage to the AcbC can produce impulsive choice in the sense of an impaired ability to choose delayed rewards [22], in addition to hyperactivity [22,24,38,41], though without impairments in attentional function [91] and without motoric impulsivity as assessed by the stop-signal task [92]. In the context of choice involving uncertain appetitive reinforcement, 'impulsivity' would equate to risk taking (less steep uncertainty discounting or greater willingness to choose unlikely rewards). AcbC lesions, however, produced a risk-averse or conservative pattern of choice in the present study. Clearly, then, AcbC-lesioned rats cannot be characterized as impulsive in all senses. A more appropriate unifying concept would seem to be that the AcbC promotes the selection, and perhaps the salience, of uncertain and delayed rewards – perhaps, in general, of rewards that are not certain, imminent, or present [58]. The AcbC promotes choice of [22] and learning with [24] delayed rewards. It appears to promote the selection of uncertain reinforcers (present results), and this is compatible with human imaging studies showing increased Acb blood flow during the selection of high-risk options [36,37]. The Acb is required for PIT, the process by which Pavlovian CSs signalling reward enhance instrumental responding for those rewards [52,53]. It is also required for autoshaping, or locomotor approach to appetitive Pavlovian CSs [38,54-57], and it influences conditioned reinforcement, the process of working for CSs previously paired with reinforcement [38,93-95]. Acb DA also contributes to subjects' motivation to work hard [96-100].

It is not known whether AcbC lesions would produce similar effects on choice involving uncertain aversive events. It would be expected that increased odds/uncertainty/probability discounting – effectively, a tendency to behave as if an uncertain outcome were *less likely *than it really is – would produce risk aversion for appetitive outcomes (reduced willingness to choose large, unlikely rewards) but risk proneness for aversive outcomes (increased willingness to choose large, uncertain punishments over small, certain punishments) [1]. In humans, at least, the delay and probability discounting processes appear similar for rewards and losses [46,101].

### Relationship to structures and neuromodulator systems innervating the AcbC

The prefrontal cortex (PFC), which projects heavily to the AcbC [102], is also involved in decision-making under conditions of uncertainty. Humans with orbitofrontal cortex (OFC) or ventromedial PFC damage are impaired on the Iowa gambling task [103-105], in which subjects must learn to differentiate between low-reward, low-risk card decks that yield a net positive outcome and high-reward, high-risk decks that yield a net negative outcome, though the precise locus and nature of the deficit seen on this task is debated [106-108]. Choice between small, likely rewards and large, unlikely rewards increases cerebral blood flow in orbital and inferior PFC [109,110], and OFC damage also impairs performance of a task requiring human subjects to choose between two possible outcomes and to bet on their choice, with lesioned subjects deciding slowly and failing to choose the optimal, most likely outcome [111]. Excitotoxic lesions of the OFC make rats less likely than sham-operated controls to choose a large, uncertain reward over a small, certain reward [112]; OFC-lesioned rats had lower indifference odds (higher indifference probabilities; steeper uncertainty discounting) and exhibited risk-averse choice, just like the AcbC-lesioned subjects in the present study. There is direct evidence that OFC lesions do alter sensitivity to the relative magnitudes of the two rewards [113], as does OFC DA depletion [114], but the effects on uncertainty discounting are present in addition to those on reinforcer magnitude sensitivity [115].

The Acb is also innervated by the a number of neuromodulator systems, including the serotonin (5-hydroxytryptamine; 5-HT) system [116]. Although manipulations of 5-HT influence choice involving delayed reinforcement, there is less evidence that they influence choice involving uncertainty and risk. Correlational studies have indicated that low cerebrospinal fluid (CSF) levels of the 5-HT metabolite 5-hydroxyindoleacetic acid (5-HIAA) are associated with risk taking in monkeys [117] and impulsive aggression, violence, and suicide in humans [118-122]. Forebrain 5-HT depletion tends to steepen temporal (delay) discounting (reviewed briefly by [28]); however, it does not appear to influence choice involving probabilistic reinforcement. Dietary tryptophan depletion [123-125] decreases levels of 5-HT metabolites in CSF, an indirect indicator of brain 5-HT levels, but has not been shown to affect probability discounting in humans [126,127]; similarly, forebrain 5-HT depletion in rats does not affect choice between small, certain rewards and large, uncertain rewards [128]. The AcbC also receives a substantial DA innervation, and DA neurons respond to reward prediction errors [18-21]. Although systemic D2-type DA receptor antagonists can induce impulsive choice involving delayed reinforcement [129], this effect may not occur in the Acb [130], the response of DA neurons specifically to reward uncertainty is debated [32-34], and little is known of the role of DA in choice involving uncertain rewards. Systemic noradrenergic (NA) blockade has also been shown to affect decision-making under uncertainty in humans, by reducing the discrimination between magnitudes of different losses when the probability of losing was high [131], though NA reuptake inhibition has not been shown to affect the Iowa gambling task [132]. However, the Acb does not receive a substantial NA innervation [133].

## Conclusion

We have shown that excitotoxic lesions of the AcbC induce risk-averse choice in rats. AcbC lesions did not prevent rats from discriminating a large reward from a small reward, or a certain reward from an uncertain reward. However, when offered the choice between a small/certain reward and a large/uncertain reward, AcbC-lesioned rats showed a reduced preference for the large/uncertain reward (compared to sham-operated controls) in their final pattern of postoperative choice. AcbC-lesioned rats exhibited a tendency to behave as if an uncertain outcome were less likely than was really the case. Together with previous studies, these results suggest that the AcbC contributes to reinforcement and choice particularly when the reinforcer is temporally distant or uncertain.

## Methods

### Subjects and housing conditions

The subjects were 24 male Lister hooded rats (Harlan-Olac UK Ltd) housed in a temperature-controlled room (minimum 22°C) under a 12:12 h reversed light-dark cycle (lights off 07:30 to 19:30). Subjects were approximately 15 weeks old on arrival at the laboratory and were given a minimum of a week to acclimatize, with free access to food, before experiments began. Preoperatively, subjects were housed in pairs; postoperatively, they were housed individually. Experiments took place between 09:00 and 21:00, with individual subjects being tested at a consistent time of day. Subjects had free access to water. During behavioural testing, subjects were fed ~15–16 g/day, an amount that maintains ~85–90% of free-feeding mass in normal male Lister hooded rats (the free-feeding mass being a steadily-increasing quantity at this age). Feeding occurred in the home cages at the end of the experimental day. As it was possible for subjects to earn substantial amounts of food in the behavioural tasks, the amount of food actually earned was taken into account when feeding with the maintenance diet in the home cages. All procedures were subject to UK Home Office approval (Project Licence 80/1767) under the Animals (Scientific Procedures) Act 1986.

### Behavioural apparatus

Behavioural testing was conducted in one of two types of operant chamber of identical configuration (from Med Associates Inc., Georgia, Vermont, USA, or Paul Fray Ltd, Cambridge, UK). Each chamber was fitted with a 2.8 W overhead house light and two retractable levers on either side of an alcove fitted with an infrared photodiode to detect head entry and a 2.8 W lightbulb ('traylight'). Sucrose pellets (45 mg, Rodent Diet Formula P, Noyes, Lancaster, New Hampshire, USA) could be delivered into the alcove. The chambers were enclosed within sound-attenuating boxes fitted with fans to provide air circulation. The apparatus was controlled by software written by RNC in C++ [134] using the Whisker control system [135]. Equal numbers of subjects were trained in the two brands of operant chamber (12 in each type). Individual subjects were always tested in the same operant chamber.

### Initial training

Rats were first trained to press the left lever for single pellets on a fixed-ratio-1 schedule, in 30-min sessions, until they had obtained a total of 100 pellets. This procedure was repeated for the right lever. They were then trained to nosepoke to initiate presentation of a lever in discrete trials. Each session began with the levers retracted and the operant chamber in darkness. Every 40 s, a trial began with illumination of the houselight and the traylight. The subject was required to make a nosepoke response within 10 s, or the current trial was aborted and the chamber returned to darkness. If the subject nosepoked within this time limit, the traylight was extinguished and a single lever presented. If the rat failed to respond on the lever within 10 s, the lever was retracted and the chamber darkened, but if it responded, the houselight was switched off, a single pellet was delivered immediately and the traylight was illuminated until the rat collected the pellet (or a 10-s collection time limit elapsed, whereupon the chamber was darkened). In every pair of trials, the left lever was presented once and the right lever once, though the order within the pair of trials was random. Rats were trained to a criterion of 60 successful trials in one hour (the maximum possible with a 40-s period being 90). They then proceeded to the full task.

### Probabilistic choice task

The task was based on delayed reinforcement choice tasks that have been described before [73,74]. The session began in darkness with the levers retracted; this was designated the intertrial state. Trials began at 40-s intervals; the format of a single trial is shown in Figure 2. Each trial began with the illumination of the houselight and the traylight. The rat was required to make a nosepoke response, ensuring that it was centrally located at the start of the trial (latency to poke was designated the initiation latency). If the rat did not respond within 10 s of the start of the trial, the operant chamber was reset to the intertrial state until the next trial began and the trial was scored as an omission. If the rat was already nosepoking when the trial began, the next stage followed immediately. Upon a successful nosepoke, the traylight was extinguished and one or both levers were extended. One lever was designated the Large/Uncertain lever, the other the Small/Certain lever (counterbalanced left/right). The latency to choose a lever was recorded. (If the rat did not respond within 10 s of lever presentation, the chamber was reset to the intertrial state until the next trial and the trial was scored as an omission.) When a lever was chosen, both levers were retracted and the houselight was switched off. Choice of the Small lever caused the certain delivery of one pellet; choice of the Large lever caused the delivery of 4 pellets with a particular probability (see below). When reinforcement was delivered, the traylight was switched on. Multiple pellets were delivered 0.5 s apart. If the rat collected the pellets before the next trial began, then the traylight was switched off and the time from delivery of the first pellet until a nosepoke occurred was recorded as the collection latency. If the rat did not collect the food within 10 s of its delivery, the operant chamber entered the intertrial state, though collection latencies were still recorded up to the start of the next trial. The chamber was then in the intertrial state and remained so until the next trial. There was no mechanism to remove uneaten pellets, but failure to collect the reward was an extremely rare event. The large-reinforcer probability was varied systematically across the session as follows. A session consisted of 5 blocks, each comprising 16 trials in which only one lever was presented (8 trials for each lever, randomized in pairs) followed by 10 free-choice trials. The probability that the large reinforcer was delivered, given that the Large lever had been chosen (*p*_reinforcer_), varied across blocks: it was initially 1, 0.5, 0.25, 0.125, and 0.0625, respectively, for each block. As trials began every 40 s and there were 130 trials per session, the total session length was ~87 minutes; subjects received one session per day. Choice ratios (percentage choice of the large reinforcer, for each trial block) were calculated using only choice trials on which the subject responded.

### Excitotoxic lesions of the AcbC

Subjects were anaesthetized with Avertin (2% w/v 2,2,2-tribromoethanol, 1% w/v 2-methylbutan-2-ol, and 8% v/v ethanol in phosphate-buffered saline, sterilized by filtration, 10 ml/kg intraperitoneally) and placed in a Kopf or Stoelting stereotaxic frame (David Kopf Instruments, Tujunga, California, USA; Stoelting Co., Wood Dale, Illinois, USA) fitted with atraumatic ear bars. The skull was exposed and a dental drill was used to remove the bone directly above the injection sites. The dura mater was broken with the tip of a hypodermic needle, avoiding damage to underlying venous sinuses. Excitotoxic lesions of the AcbC were made by injecting 0.5 *μ*l of 0.09 M quinolinic acid (Sigma, UK) per side through a glass micropipette at coordinates 1.2 mm anterior to bregma, ± 1.8 mm from the midline, and 7.1 mm below the skull surface at bregma; the incisor bar was 3.3 mm below the interaural line [136]. The toxin had been dissolved in 0.1 M phosphate buffer (composition 0.07 M Na_2_HPO_4_, 0.028 M NaH_2_PO_4 _in double-distilled water, sterilized by filtration) and adjusted with NaOH to a final pH of 7.2–7.4. Toxin was injected over 3 min and the micropipette was left in place for 2 min following injections. Sham lesions were made in the same manner except that vehicle was infused. At the end of the operation, animals were given 15 ml/kg of sterile 5% w/v glucose, 0.9% w/v sodium chloride intraperitoneally. They were given a week to recover, with free access to food, and were handled regularly. Any instances of postoperative constipation were treated with liquid paraffin orally and rectally. At the end of this period, food restriction commenced or was resumed.

### Postoperative testing

Subjects were trained preoperatively and tested postoperatively according to the schedule shown in Table 1. In the basic task, used for preoperative training, the probability of large reinforcer delivery declined across trial blocks from 1 to 0.0625 (in the order 1, 0.5, 0.25, 0.125, 0.0625). After subjects had been tested postoperatively for 12 sessions on this schedule, satiety tests were given, to establish the effect of varying primary motivational state on preference for probabilistic reinforcement. Subjects were tested for 4 sessions while alternating between hungry and sated states on consecutive days in counterbalanced fashion (half the subjects experienced hungry and sated days in the order HSHS, and half in the order SHSH). Following a 'hungry' session, animals were placed on free food (maintenance diet) until the start of the next day's 'sated' session, at which time the food was again removed for the 'hungry' session to follow. The comparison was therefore between food deprivation for ~22 h and satiety. Next, subjects were returned to the hungry state and tested for 6 sessions on a schedule in which both the large and small reinforcer were delivered with certainty. Next, the element of uncertainty was reintroduced for another 12 sessions, but this time the probability of large reinforcer delivery (given that the Large lever had been chosen) *increased *across blocks from 0.0625 to 1 (in the order 0.0625, 0.125, 0.25, 0.5, 1). Finally, subjects were tested for 6 sessions with the large reinforcer always being very unlikely (*p *= 0.0625), with the small reinforcer remaining certain.

### Locomotor activity in a novel environment

Locomotor activity was measured in wire mesh cages, 25 (W) × 40 (D) × 18 (H) cm, each equipped with a water bottle and two horizontal photocell beams situated 1 cm from the floor that enabled movements along the long axis of the cage to be registered. Subjects were placed in these cages, which were initially unfamiliar to them, and their activity was recorded for 2 h. All animals were tested in the food-deprived state. Locomotor hyperactivity and reduced body mass gain have previously been part of the phenotype of AcbC-lesioned rats, though without alterations in the consumption of the reinforcer used in the present experiments [22,24,38,39,41].

### Histology

Rats were deeply anaesthetized with pentobarbitone sodium (200 mg/ml, minimum of 1.5 ml i.p.) and perfused transcardially with 0.01 M phosphate-buffered saline (PBS) followed by 4% paraformaldehyde in PBS. Their brains were removed and postfixed in paraformaldehyde before being dehydrated in 20% sucrose for cryoprotection. The brains were sectioned coronally at 60 *μ*m thickness on a freezing microtome and every third section mounted on chromium potassium sulphate/gelatin-coated glass microscope slides and allowed to dry. Sections were passed through a series of ethanol solutions of descending concentration (3 minutes in each of 100%, 95%, and 70% v/v ethanol in water) and stained for ~5 min with cresyl violet. The stain comprises 0.05% w/v aqueous cresyl violet (Raymond A. Lamb Ltd, Eastbourne, UK), 2 mM acetic acid, and 5 mM formic acid in water. Following staining, sections were rinsed in water and 70% ethanol before being differentiated in 95% ethanol. Finally, they were dehydrated and delipidated in 100% ethanol and Histoclear (National Diagnostics, UK) before being cover-slipped using DePeX mounting medium (BDH, UK) and allowed to dry. The sections were used to verify lesion placement and assess the extent of lesion-induced neuronal loss. Lesions were detectable as the absence of visible neurons (cell bodies of the order of 100 *μ*m in diameter with a characteristic shape and appearance), often associated with a degree of tissue collapse (sometimes with consequent ventricular expansion when the lesion was adjacent to a ventricle) and gliosis (visible as the presence of smaller, densely-staining cells).

### Data analysis

Data collected by the chamber control programs were imported into a relational database (Microsoft Access 97) for case selection and analysed with SPSS 11. Figures were created with SigmaPlot 2001/v7 and Adobe Illustrator 8. All graphs show group means and error bars are ±1 standard error of the mean (SEM) unless otherwise stated. Count data (e.g. locomotor activity counts), for which variance increases with the mean, were subjected to a square-root transformation prior to any analysis [137]. Homogeneity of variance was verified using Levene's test [138]. General linear models are described as *dependent variable *= *A*_2 _× *B*_*cov *_× (*C*_5 _× *D*_*cov *_× *S*) where A is a between-subjects factor with two levels, B is a between-subjects covariate, C is a within-subjects factor with five levels, and D is a within-subjects covariate; S denotes subjects in designs involving within-subjects factors [139]. For repeated measures analyses, Mauchly's test of sphericity of the covariance matrix was applied [140] and the degrees of freedom corrected to more conservative values by multiplying them by the Huynh-Feldt epsilon 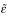 for any terms involving factors in which the sphericity assumption was violated [141]. Where multiple comparisons were conducted *post hoc *following a significant overall ANOVA effect for a factor with more than three levels, *p *values were corrected using the Šidák correction [142], in which *p*_corrected _= 1 - (1 - *p*_uncorrected_)^*n *^for *n *comparisons.

## List of abbreviations used

5-HIAA, 5-hydroxyindoleacetic acid

5-HT, 5-hydroxytryptamine (serotonin)

Acb, nucleus accumbens

AcbC, nucleus accumbens core

AcbSh, nucleus accumbens shell

ANOVA, analysis of variance

CS, conditioned stimulus

CSF, cerebrospinal fluid

DA, dopamine


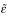
, Huynh-Feldt epsilon

h, hour

i.p., intraperitoneal

min, minute

NA, noradrenaline

OFC, orbitofrontal cortex

*p*, probability

*p*_reinforcer_, probability of delivery of the large reinforcer after it had been chosen

*p*_statistical_, statistical *p *value (probability of obtaining the observed data, or results more extreme, were the null hypothesis to be true)

PBS, phosphate-buffered saline

PIT, Pavlovian-instrumental transfer

PFC, prefrontal cortex

*r*^2^, proportion of variance explained

SED, standard error of the difference between means

SEM, standard error of the mean

SS, sum of squares (sum of squared deviations)

v/v, volume per unit volume

w/v, weight per unit volume

[*x*, *y*), a range that includes *x *but not *y*

## Authors' contributions

RNC conceived and designed the studies, supervised NJH, wrote the software, performed the surgery, and drafted the manuscript. NJH participated in the design of the studies, and tested the animals. The work contributed to NJH's B.A. degree. Both authors analysed the results, and read and approved the final manuscript.

## Figures and Tables

**Table 1:** Testing schedule for probabilistic choice task. Subjects were trained and tested according to the schedule shown here. Initial pre- and postoperative testing was conducted with the probability of large reinforcer delivery declining across trial blocks from 1 to 0.0625 (the steps were *p *= 1, 0.5, 0.25, 0.125, and 0.0625). Subsequently, subjects were tested alternating between the hungry and sated state (as described in the Methods), before the reinforcement probabilities were manipulated further, as indicated.

Sessions	Description	Probability (small reward of 1 pellet)	Probability (large reward of 4 pellets)
1–12	Preoperative training (12 sessions)	*p *= 1	*p *= 1 to 0.0625, decreasing
-	Surgery and recovery	-	-
13–24	Postoperative baseline testing (12 sessions)	*p *= 1	*p *= 1 to 0.0625, decreasing
25–28	Satiety tests (4 sessions)	*p *= 1	*p *= 1 to 0.0625, decreasing
29–34	Equal probabilities, certain (6 sessions)	*p *= 1	*p *= 1
35–46	Increasing probabilities (12 sessions)	*p *= 1	*p *= 0.0625 to 1, increasing
47–52	Large reinforcer always very unlikely (6 sessions)	*p *= 1	*p *= 0.0625
